# Impact of dietary plant flavonoids on 7,8‐dihydroxyflavone transepithelial transport in human intestinal Caco‐2 cells

**DOI:** 10.1002/fsn3.3581

**Published:** 2023-07-30

**Authors:** Yufeng Chen, Guobin Xia, Chunfeng Wang, Huawei Wu, Xiaogang Xu, Genxiang Mao, Jiong Wu, Zhenlei Zhao

**Affiliations:** ^1^ Department of Food Science and Nutrition, School of Biosystems Engineering and Food Science, Zhejiang Key Laboratory for Agro‐Food Processing, Zhejiang Engineering Center for Food Technology and Equipment Zhejiang University Hangzhou China; ^2^ Zhejiang Provincial Key Lab of Geriatrics and Geriatrics Institute of Zhejiang Province, Department of Geriatrics Zhejiang Hospital Hangzhou China; ^3^ Ningbo Today Food Co Ltd Ningbo China; ^4^ Section of Neonatology, Department of Pediatrics Baylor College of Medicine Houston Texas USA

**Keywords:** 7,8‐Dihydroxyflavone, Caco‐2 cell, Kaempferol, P‐glycoprotein, quercetin, Transepithelial transport

## Abstract

7,8‐dihydroxyflavone (7,8‐DHF) is a biologically active flavone with various physiological activities, including neuroprotection, anti‐inflammation, and weight loss. Previous studies have found that the efflux protein P‐glycoprotein (P‐gp) significantly affects the transepithelial transport of 7,8‐DHF in the intestine, resulting in its low oral bioavailability. Based on this, in this study, a Caco‐2 monolayer cell model was used to investigate 14 dietary plant flavonoids as potential P‐gp inhibitors, and their effects on the transepithelial transport and in vitro digestion of 7,8‐DHF were explored. The results showed that among the 14 plant flavonoids, hesperetin, epigallocatechin gallate, fisetin, kaempferol, quercetin, and isoorientin increased and the apparent permeability coefficients (*P*_app_) of 7,8‐DHF at AP → BL direction and lowered *P*_app_ value at BL → AP direction to varying degrees, reducing the efflux ratio of 7,8‐DHF less than 1.5. In particular, kaempferol and quercetin exhibited the best effect on promoting the transepithelial transport of 7,8‐DHF, especially when used at molar concentration ratios of 1:1 and 1:2 with 7,8‐DHF. This is beneficial for improving the oral bioavailability of 7,8‐DHF. Meanwhile, 7,8‐DHF was found to maintain structural stability in simulated saliva, gastric juice, and intestinal juice, and its stability was not affected by the coexistence of quercetin and kaempferol. Overall, this study provided a theoretical basis for seeking natural and safe P‐gp inhibitors to improve the oral absorption of natural products.

## Graphical abstract

Kaempferol as a P‐gp inhibitor could boost 7,8‐DHF transport in Caco‐2 cell. Quercetin as a P‐gp inhibitor could promote 7,8‐DHF transport in Caco‐2 cell. Kaempferol and quercetin had no significant effects on structural stability of 7,8‐DHF in vitro digestion.

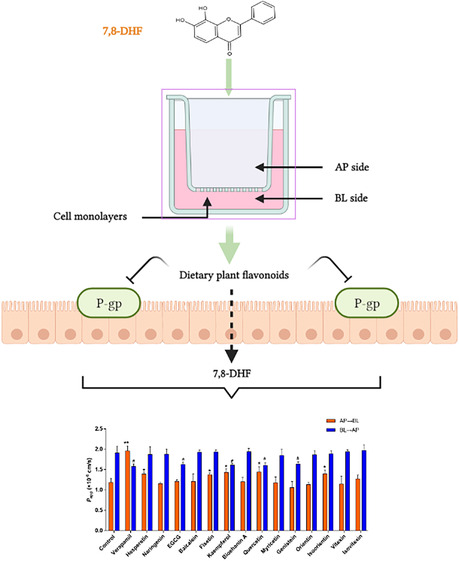

## 1 INTRODUCTION

7,8‐dihydroxyflavone (7,8‐DHF) is a rare flavonoid compound found in nature, specifically in *Godmania aesculifolia*, *Tridax procumbens*, *Primula*, and *Malus hupehensis* (Chen et al., 2022; Colombo et al., 2014). Numerous studies have demonstrated that 7,8‐DHFfunctions as a small molecule agonist of the tropomyosin‐related kinase B (TrkB), effectively simulating the function of brain‐derived neurotrophic factor (BDNF) and activating downstream signaling pathways, such as mitogen‐activated protein kinase/extracellular signal‐regulated kinase (MAPK/ERK), phosphatidylinositol 3‐kinase/protein kinase B (PI3K/Akt), and phospholipase Cγ1/protein kinase C (PLCγ1/PKC; Fang, Cao, et al., 2022; Fang, Luo, et al., 2022; Jang et al., 2010; Sharma et al., 2023). Among them, the activation of MAPK/ERK pathway can stimulate neural differentiation, PI3K/Akt pathway can promote neuron survival and growth, while PLCγ1/PKC signaling is crucial for synaptic plasticity. Currently, 7,8‐DHF is being studied for its potential preventative and therapeutic applications in various BDNF/TrkB signal‐related diseases, including Alzheimer's disease, Parkinson's disease, Huntington's disease, Rett syndrome, depression, and metabolic syndrome (Chen et al., 2018, 2021; Paul et al., 2021; Yang & Zhu, 2022; Zhao et al., 2021). However, natural plant flavonoids generally have low oral bioavailability. The adjacent dihydroxy in the A‐ring of 7,8‐DHF is prone to methylation, glycosylation, and sulfation in the liver, so its oral bioavailability in animal experiments (C57BL/6 mice) is only 4.6% (Liu et al., 2013). This limits its potential application in the development of functional food factors according to principles of food science and engineering.

Previously, we investigated the transepithelial transport mechanism of 7,8‐DHF using a Caco‐2 monolayer cell model. Our findings revealed that 7,8‐DHF can be transported across the membrane by both influx and efflux transporters (Chen et al., 2019). However, the involvement of efflux transporters, such as P‐glycoprotein (P‐gp) and multi‐drug resistance‐associated proteins (MRPs), significantly reduced the transmembrane transport efficiency of 7,8‐DHF, with P‐gp having a stronger impact than MRPs. In the intestine, P‐gp is highly expressed at the apical brush border of enterocytes and plays a critical role in removing some exogenous or endogenous substances from cells to protect the body. Nevertheless, this also negatively affects the absorption of certain drugs or functional factors, resulting in a decreased bioavailability (Azman et al., 2022; Ernst et al., 2010; Wang et al., 2023). Currently available P‐gp inhibitors are synthetic compounds, including verapamil, diltiazem, and cyclosporine A, which may cause immunosuppressive and cardiovascular side effects (Nguyen et al., 2021; Tsuruo et al., 1981). Alternatively, natural P‐gp inhibitors, such as flavonoids, coumarins, terpenes, and alkaloids (Abuznait et al., 2011; Fang, Cao, et al., 2022; Fang, Luo, et al., 2022; Lei et al., 2013; Shah et al., 2023), have high food safety and wider application prospects in the functional food and drug field. For instance, *Schutte* et al. reported that 20 and 40 μmol/L of quercetin can increase the apparent permeability coefficient of 2‐amino‐1‐methyl‐6‐phenylimidazo[4,5b]pyridine (PhIP) and improve its oral bioavailability by inhibiting the expression of P‐gp (Schutte et al., 2008). Similarly, *Ravindra Babu* et al. found that quercetin, as a typical P‐gp and cytochrome P450 inhibitor, respectively improves the oral bioavailability of etoposide (an anticancer drug) and ranolazine (an anti‐anginal drug) in rats (Babu et al., 2013). Additionally, *Li* et al. showed that the oral bioavailability of diazepam in rats was significantly enhanced and the half‐life was prolonged in the presence of epigallocatechin gallate (EGCG) at doses of 4 and 12 mg/kg.bw, suggesting that their mechanism of action involves the inhibition of P‐gp and CYP3A activity (Li & Choi, 2008). Furthermore, grapefruit flavonoids and candesartan cilexetil were co‐administered in a certain proportion to improve the oral bioavailability of candesartan cilexetil in rats (Gurunath et al., 2015).

In this study, we used an in vitro Caco‐2 monolayer cell model to investigate the effect of 14 common plant flavonoids, including hesperetin, naringenin, EGCG, baicalein, fisetin, kaempferol, biochanin A, quercetin, myricetin, genistein, orientin, isoorientin, vitexin, and isovitexin as natural P‐gp inhibitors to promote the transepithelial transport of 7,8‐DHF. Most of the 14 common plant flavonoids, which encompass various structural types of flavonoid compounds, including flavone glycosides, flavanols, flavanes, and flavonoid glycoside, have been previously reported in the literature to potentially exhibit inhibitory effects on P‐gp. However, it is unknown whether they have any role in promoting the transport of 7,8‐DHF as P‐gp inhibitor. Additionally, the impact of these inhibitors on the in vitro digestion of 7,8‐DHF was investigated. This research provides useful information for improving the intestinal absorption of 7,8‐DHF and other natural products.

## 2 MATERIALS AND METHODS

### 2.1 Materials and chemicals

7,8‐Dihydroxyflavone (purity = 98%) was purchased from Tokyo Chemical Industry Co., Ltd. Verapamil hydrochloride (purity = 99%), hesperetin (purity = 98%), naringenin (purity = 98%), baicalein (purity = 98%), fisetin (purity = 98%), kaempferol (purity = 98%), biochanin A (purity = 98%), quercetin (purity = 98%), genistein (purity = 98%) and α‐amylase (100,000 U/g) were obtained from Aladdin. Orientin (purity = 98%), isoorientin (purity = 98%), vitexin (purity = 98%), and isovitexin (purity = 98%) were purchased from Chengdu Pufei De Biotech Co., Ltd. Pepsin (≥10,000 NFU/mg), bile salts, ethylene glycol tetraacetic acid and cell counting kit‐8 (CCK‐8) were obtained from Shanghai Sangon Biotech. Pancreatin (4× USP specifications) were obtained from Sigma‐Aldrich. Dulbecco's modified Eagle's medium (DMEM, with 4.5 g/L glucose, L‐glutamine and sodium pyruvate) and porous polycarbonate membrane Transwell inserts (0.4 μm pore size and 12 mm diameter) were purchased from Corning Incorporated. HPLC‐grade methanol was obtained from TEDIA. An antibiotic solution (10,000 IU/mL penicillin, 10,000 μg/mL streptomycin), a non‐essential amino acid solution, Hank's balanced salt solution (HBSS), and 0.25% trypsin‐ethylenediaminetetraacetic acid solution were purchased from Solarbio. Heat‐inactivated fetal bovine serum (FBS) was obtained from Gibco Laboratory. Other chemicals and reagents of analytical grade were purchased from Sinopharm Chemical Reagent Co.

### 2.2 Cell culture and its monolayer cell model establishment

The Caco‐2 cells, within passages 40~60, were acquired from the Shanghai Cell Bank of the Chinese Academy of Sciences. To culture the Caco‐2 cells, DMEM high glucose medium supplemented with 10% FBS, 1% antibiotic solution, and 1% non‐essential amino acid solution was used. The cells were cultured in a CO_2_ incubator (SPX150, Hangzhou Sanhe Innovation Technology Co., Ltd) at 37°C with a gas mixture of 95% O_2_ and 5% CO_2_. The medium was renewed every 1~2 days and when the Caco‐2 cells reached 80% confluence, they were passaged using 0.25% trypsin every 3~4 days at a ratio of 1:3 (Chen, Xia, et al., 2020; Chen, Zhao, et al., 2020).

To obtain differentiated monolayer cell membranes, Caco‐2 cells were seeded at a density of 5 × 10^4^ cells/cm^2^ in 12‐well Transwell plates, observed by an inverted light microscope (CKX41GF, Olympus). The medium was changed every 2 days for the first 2 weeks and then daily until 21~28 days. The integrity of the monolayer structure was evaluated by measuring transepithelial electrical resistance (TEER) using a Millicell ERS electrode (Millipore Corp). Before measurement, the electrode was disinfected in 70% alcohol for 15 min and then equilibrated in HBSS for 30 min. To balance, preheated HBSS solution (37°C) was added to the apical (AP) and basolateral (BL) sides on the Transwell plate for 15 min. The electrode was then inserted to measure resistance (*R*) value, while the blank R_0_ value was measured simultaneously. TEER was calculated using the following formula:

(1) $$\text{TEER}=\left(R-R_{0}\right)\times A$$

where TEER is the transepithelial electrical resistance value (Ω cm^2^), *R*_0_ and *R* are the resistance values of the blank pore and cell pore (Ω), and *A* is the area of the cell monolayer membrane (1.12 cm^2^). TEER >300 Ω cm^2^ was considered as a compact and complete monolayer membrane (Steenson et al., 2022).

### 2.3 Cytotoxicity assay

To determine the cytotoxicity of 7,8‐DHF for transport experiments, a CCK‐8 assay was carried out (Han et al., 2023). A total of 200 μL culture medium containing Caco‐2 cells at a density of 3 × 10^4^ cells/mL was seeded in a 96‐well plate and incubated for 24 h to allow for adherence. After the incubation period, the culture medium was removed and replaced with 200 μL fresh medium containing 7,8‐DHF at concentrations of 1, 10, 50, 100, and 300 μmol/L. Additionally, 200 μL medium without 7,8‐DHF was added as the control group, while medium without cells was used as the blank group. Following 24 h of drug stimulation, 10 μL CCK‐8 reagent was added to each well followed by another 2 h incubation. The absorbance was measured at 450 nm wavelength using an enzyme‐linked immunosorbent assay reader (Eonmei, BioTek). Each sample was repeated five times, and the cell survival rate was calculated using the following formula:

(2) $$\text{Cell survival rate}\;\left(\%\right)=\frac{{\mathrm{OD}}_{\text{sample group}}-{\mathrm{OD}}_{\text{blank group}}}{{\mathrm{OD}}_{\text{control group}}-{\mathrm{OD}}_{\text{blank group}}}\times 100$$

where OD represents the absorbance of each sample at 450 nm wavelength.

### 2.4 Transepithelial transport experiments for screening P‐gp inhibitors of plant flavonoids

For this experiment, the 7,8‐DHF sample was first dissolved in a small amount of dimethyl sulfoxide (DMSO) and then diluted with HBSS to a working concentration of 10 μmol/L. The final DMSO concentration in the working solution was controlled to be less than 0.5%. Using the Caco‐2 monolayer cell model, a natural P‐gp inhibitor that could enhance the transepithelial transport of 7,8‐DHF was screened from 14 flavonoids (hesperetin, naringenin, EGCG, baicalein, fisetin, kaempferol, biochanin A, quercetin, myricetin, genistein, orientin, isoorientin, vitexin, and isovitexin). Flavonoid samples were added to the AP and BL sides at equimolar concentrations with 7,8‐DHF (the sample concentration in HBSS solution was 10 μmol/L). Prior to transport, dietary flavonoids were pretreated on the AP side for 30 min. Then, these flavonoid reagents were removed, and a 0.5 mL HBSS solution containing various dietary flavonoids with 7,8‐DHF (concentration of 10 μmol/L) was added to the AP side, while 1.5 mL of blank HBSS was added to the BL side. Conversely, 1.5 mL of HBSS solution containing various dietary flavonoids with 7,8‐DHF (10 μmol/L) was added to the BL side, and 0.5 mL of blank HBSS was added to the AP side. Following 150 min of transport, 200 μL of acceptor solution was taken from the receiving side for UPLC determination. Verapamil (A P‐gp inhibitor, 100 μmol/L) was used as a positive control.

The TEER values of the cell monolayers were measured before and after transport experiments to characterize the integrity of monolayers.

The magnitude of the apparent permeability coefficients (*P*_app_, cm/s) and efflux ratio (ER) reflects the ability of the test substance to pass through the monolayer cells and the speed of absorption.

The *P*_app_ and ER were calculated according to following equation (Da Silva Santos et al., 2022; Song et al., 2022):

(3) $$P_{\mathrm{app}}\;\left(\mathrm{cm}/s\right)=\left(dQ/dt\right)\;\left(1/{\mathrm{AC}}_{0}\right)$$

where d*Q*/d*t* is the transport rate on the receiver compartment (μmol/L/s), *A* is the membrane surface area (1.12 cm^2^), and *C*_0_ is the initial concentration in the donor compartment (μmol/L).

(4) $$\mathrm{ER}=\frac{P_{\mathrm{app}\;\mathrm{AB}}}{P_{\mathrm{app}\;\mathrm{BA}}}$$

where *P*_app AB_ is the apparent permeability coefficient for AP → BL transport, and *P*_app BA_ is the apparent permeability coefficient for BL → AP transport.

Effect of different concentrations of specific flavonoids on 7,8‐DHF transepithelial transport was investigated. The operating procedure was followed by above section, with the exception that specific dietary flavonoid samples were added to the AP and BL sides at varying molar ratios with 7,8‐DHF (2:1, 1:1, 1:2, and 1:4). The specific dietary flavonoids were dissolved in HBSS while controlling the DMSO concentration to be less than 0.5%. After 150 min of transport, 200 μL of acceptor solution was taken from the receiving side and 7,8‐DHF content was determined using UPLC.

After the 150 min transepithelial transport experiment, the 7,8‐DHF content in Caco‐2 cells was determined (Wu et al., 2021). The cell surface was rapidly washed three times with ice‐cold HBSS solution. The cells were harvested using a cell scraper and resuspended in 1.0 mL of 0.1% extraction solution (phosphate: methanol = 1:1, v/v) before being homogenized in an ultrasonicator. After centrifugation at 4°C and 11,000 × *g* for 15 min, the supernatant was collected and the protein concentration in Caco‐2 cells was determined using a BSA assay kit.

### 2.5 Stability of 7,8‐DHF in HBSS and Caco‐2 cells

To assess the stability of 7,8‐DHF in HBSS and the metabolic behavior of enzymes toward 7,8‐DHF in Caco‐2 cells (Chen, Xia, et al., 2020; Chen, Zhao, et al., 2020), a 10 μmol/L 7,8‐DHF sample was prepared in HBSS (pH = 7.4), then incubated in a constant temperature shaker at (37 ± 1)°C and 50 rpm for 60 and 240 min. Simultaneously, 0.5 mL of the aforementioned solution was added to the AP side of the Transwell culture plate containing Caco‐2 cells, while 1.5 mL of blank HBSS added to the BL side. The Transwell culture plate was placed in the constant temperature shaker and cultured for 60 and 240 min, and all solutions from the AP and BL sides were collected. The Caco‐2 cell stability test was performed under both inhibitory and non‐inhibitory conditions, and the 7,8‐DHF content in the samples was determined.

### 2.6 Evaluation of the in vitro digestion stability of 7,8‐DHF

The in vitro digestion model proposed by *Versantvoort* et al.'s report was modified as follows (Versantvoort et al., 2005): A 1 mL solution of 7,8‐DHF and a 1 mL solution of 7,8‐DHF complexed with different P‐gp inhibitors (concentration of 7,8‐DHF was 100 μmol/L) were added to 3 mL simulated saliva containing α‐amylase at pH 6.8 ± 0.2, and digested for 5 min using a water bath magnetic stirrer at 37°C. The mixed saliva was subsequently added to 6 mL simulated gastric fluid containing pepsin at pH = 2.5 and digested for 2 h under magnetic stirring. The pH of the system was then adjusted to 7.5 and simulated intestinal fluid containing pancreatin and bile salts was added for 2 h of stirring. During the process, a certain amount of the digested liquid sample from the simulated saliva, gastric fluid, and intestinal fluid were taken, and ethanol was added to precipitate and inactivate the enzymes. After removing excess water and ethanol by rotary evaporation at 40°C, 5 mL of methanol was added to dissolve the samples. A 1 mL aliquot was placed in a high‐speed centrifuge tube (3K15, Sigma) and centrifuged at 4°C, 11000 × *g* for 15 min. The supernatant was used to determine 7,8‐DHF content using UPLC.

### 2.7 UPLC analysis

A volume of 200 μL of receiving solution was transferred into a 1 mL centrifuge tube, followed by adding 10 μL of internal standard solution (10 μg/mL apigenin) and 200 μL of methanol. The mixture was vortexed for 2 min and then centrifuged at 4°C and 11,000 × *g* for 15 min. Thereafter, 5 μL of the supernatant was injected into the UPLC system. The UPLC system used in this study was a Waters ACQUITY UPLC H‐CLASS series instrument (Waters) equipped with a quaternary solvent manager, a sample manager‐flow through needle, a chromatographic column heater and a PDA eλ detector. The UPLC system conditions were based on our previous methods (Chen, Xia, et al., 2020; Chen, Zhao, et al., 2020): the chromatographic column was C18 with a particle size of 1.7 μm and dimension of 2.1 mm × 50 mm. The mobile phase comprised of methanol (solvent A) and 0.05% trifluoroacetic acid of ultrapure water (solvent B). The gradient elution program was as follows: 20% solvent A (0~1 min), 20%~80% solvent A (1~5 min), 80%~100% solvent A (5~7 min), 100 ~ 20% solvent A (7~8 min), and 20% solvent A (8~9 min). The flow rate maintained at 0.2 mL/min, the temperature was set to 37°C, and the PDA detector wavelength was set at 330 nm.

### 2.8 Statistical analysis

All experiments were performed with a minimum of three replicates. Data analysis was conducted using GraphPad Prism 6.0 software (GraphPad Software), and presented as mean ± standard deviation (SD). One‐way analysis of variance (ANOVA) followed by Tukey's post hoc test was employed to compare all groups, using an unpaired two‐tailed Student's *t*‐test distribution. A *p*‐value of less than .05 was considered to indicate a statistically significant difference between the groups.

## 3 RESULTS AND DISCUSSION

### 3.1 Cellular integrity and cytotoxicity of Caco‐2 monolayer cell

**FIGURE 1:**
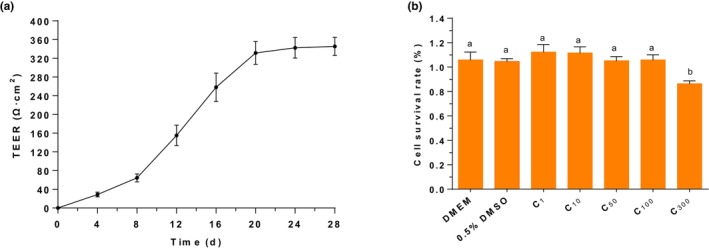
(a) TEER value of the Caco‐2 cell monolayers during culture. Data were shown as mean ± SD (*n* = 12 per group). (b) Cytotoxicity of 7,8‐DHF on Caco‐2 cells as determined using the CCK‐8 assay. Data were shown as mean ± SD (*n* = 3 per group), graph bars with different letters on top correspond to statistically significant results (*p* < .05) based on one‐way ANOVA analysis followed by Tukey's honest significant difference post hoc tests.

As depicted in Figure 1a, the TEER values of Caco‐2 cells gradually increased with extended cultivation time. From the 8th day onwards, the TEER value sharply increased and stabilized after 20 days. Additionally, all TEER values of Caco‐2 cells were greater than 300 Ω cm^2^ after 20 days, consistent with those previously reports (Xiang et al., 2018; Zhou et al., 2018). These findings suggested that the density and integrity of the monolayer of Caco‐2 cells were sufficient for the transmembrane transport test of 7,8‐DHF. In addition, Figure 1b illustrates the results obtained from determining the cell toxicity of different concentrations of 7,8‐DHF (1, 10, 50, 100, and 300 μmol/L) toward Caco‐2 cells using a CCK‐8 assay. A cell viability above 90% indicates that the compounds or drugs are non‐toxic to cells at the indicated concentrations (Kunene et al., 2021). The results showed no significant cell toxicity within the concentration range of 1~100 μmol/L. However, certain cell toxicity was observed when the 7,8‐DHF concentration reached 300 μmol/L. Therefore, the subsequent transport experiments were conducted using 7,8‐DHF concentrations between 1~100 μmol/L.

### 3.2 Effect of different flavonoids on bi‐directional permeation and efflux of 7,8‐DHF in a Caco‐2 monolayer cell

**FIGURE 2:**
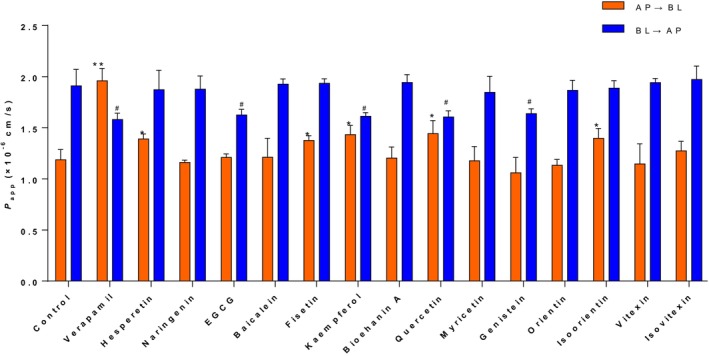
Effects of natural flavonoids on bi‐directional *P*_app_ value of 7,8‐DHF across Caco‐2 monolayers. *p < .05, **p < .01 indicate a significant difference between each group and the control group in the AP → BL direction; ^#^ *p* < .05 indicates a significant difference between each group and the control group in the BL → AP direction. Control: the control group (7,8‐DHF without inhibitor).

Figure 2 shows the effects of adding equimolar amounts of hesperetin, EGCG, fisetin, quercetin, kaempferol, genistein, and isoorientin to 7,8‐DHF solution on the *P*_app_ values of 7,8‐DHF in Caco‐2 monolayer membranes. Compared with the control group, the *P*_app_ values of 7,8‐DHF in Caco‐2 monolayer membrane AP → BL side were significantly increased (*p* < .05) after adding hesperetin, fisetin, kaempferol, quercetin, and isoorientin, with values of (1.39 ± 0.04) × 10^−6^, (1.38 ± 0.04) × 10^−6^, (1.43 ± 0.07) × 10^−6^, (1.44 ± 0.10) × 10^−6^ and (1.40 ± 0.08) × 10^−6^ cm/s, respectively (Table 1). Conversely, the *P*_app_ values of 7,8‐DHF in Caco‐2 monolayer membrane BL → AP side were significantly decreased (*p* < .05) after adding equimolar amounts of EGCG, kaempferol, quercetin, and genistein, with values of (1.63 ± 0.05) × 10^−6^, (1.61 ± 0.03) × 10^−6^, (1.61 ± 0.05) × 10^−6^ and (1.64 ± 0.04) × 10^−6^ cm/s, respectively (Table 1). This indicates that adding the above flavonoid samples effectively increased the absorption of 7,8‐DHF in Caco‐2 cells and inhibited its efflux. Among them, both kaempferol and quercetin increased the *P*_app AB_ value of 7,8‐DHF and reduced the *P*_app BA_ value, resulting in decreases in their respective ER values to 1.12 and 1.11, respectively (compared with the 1.61 ER of control group). Previous studies have reported that kaempferol and quercetin can down‐regulate multidrug‐resistance protein‐1 (MDR1) and reduce P‐gp expression (Abdallah et al., 2015; Teng et al., 2021). *Wang* et al. used an extract of *Ginkgo biloba* leaves (rich in quercetin and kaempferol) for Caco‐2 monolayer cell membrane transport experiments, demonstrating that quercetin and kaempferol regulated the expression of P‐gp transporter protein (Wang et al., 2005). *Ferreira* et al. also studied the regulatory effect of kaempferol on P‐gp transporter protein. They used rhodamine 123, which has a high affinity with P‐gp transporter protein as the research object, and found that compared with other flavonoid samples (baicalein and silybin), kaempferol could significantly increase the intracellular accumulation of rhodamine 123 within 240 min (Ferreira et al., 2018).

**TABLE 1:** Effects of natural flavonoids on bi‐directional *P*_app_ and *ER* value of 7,8‐DHF across Caco‐2 monolayers.

Group	*P*_app AB_ (×10^−6^ cm/s)	*P*_app BA_ (×10^−6^ cm/s)	ER	Modulatory effect
Control	1.19 ± 0.08	1.91 ± 0.13	1.61	/
Verapamil	1.96 ± 0.10**	1.58 ± 0.05^#^	0.81	+
Hesperetin	1.39 ± 0.04*	1.87 ± 0.16	1.35	+
Naringenin	1.16 ± 0.02	1.88 ± 0.11	1.61	−
EGCG	1.21 ± 0.03	1.62 ± 0.05^#^	1.34	+
Baicalein	1.21 ± 0.15	1.93 ± 0.04	1.59	+
Fisetin	1.37 ± 0.04*	1.93 ± 0.04	1.41	+
Kaempferol	1.43 ± 0.07*	1.61 ± 0.03^#^	1.12	+
Biochanin A	1.20 ± 0.09	1.94 ± 0.06	1.62	−
Quercetin	1.44 ± 0.10*	1.60 ± 0.05^#^	1.11	+
Myricetin	1.18 ± 0.11	1.85 ± 0.13	1.57	+
Genistein	1.06 ± 0.12	1.64 ± 0.04^#^	1.55	+
Orientin	1.13 ± 0.05	1.86 ± 0.08	1.65	−
Isoorientin	1.40 ± 0.08*	1.89 ± 0.06	1.35	+
Vitexin	1.14 ± 0.16	1.94 ± 0.03	1.70	−
Isovitexin	1.27 ± 0.08	1.97 ± 0.11	1.55	+

*p < .05, **p < .01 indicates a significant difference between each group and the control group in the AP → BL direction; ^#^ *p* < .05 indicates a significant difference between each group and the control group in the BL → AP direction. + indicates that the inhibitor has a significant effect on 7,8‐DHF transport. − indicates that the inhibitor has no significant effect on 7,8‐DHF transport.

### 3.3 Effect of different flavonoids on the uptake amount of 7,8‐DHF in Caco‐2 cells

**FIGURE 3:**
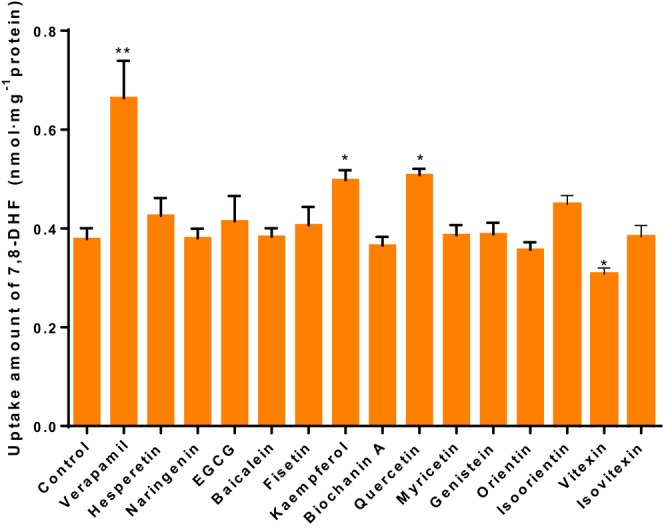
Uptake amount of 7,8‐DHF in the Caco‐2 cells at the end of transport time with various flavonoids (nmol·mg^−1^ protein). *p < .05, **p < .01, versus Control, the significant differences were analyzed by unpaired two‐tailed Student's *t*‐test.

At the end of the transport of 7,8‐DHF involving various P‐gp inhibitors of flavonoids, we determined the amount of 7,8‐DHF remaining in the cells (Figure 3). Treatment with the P‐gp inhibitor verapamil led to a significant increase (*p* < .01) in the uptake of 7,8‐DHF compared with the control group. Conversely, treatment with vitexin resulted in a significant decrease in the uptake of 7,8‐DHF compared with the control group (*p* < .05). Moreover, after treatment with kaempferol and quercetin, the uptake amount of 7,8‐DHF in Caco‐2 cells was increased from (0.378 ± 0.018) nmol/mg protein to (0.498 ± 0.016) and (0.508 ± 0.011) nmol/mg protein, respectively. Although there was no significant increase observed with a P‐gp specific inhibitor, there was still a significant difference in increase (*p* < .05). These results were consistent with those for the cumulative transport amount of 7,8‐DHF across Caco‐2.

### 3.4 Effect of different concentrations of flavonoids on bi‐directional permeation, efflux, and uptake amount of 7,8‐DHF in a Caco‐2 monolayer cell

**FIGURE 4:**
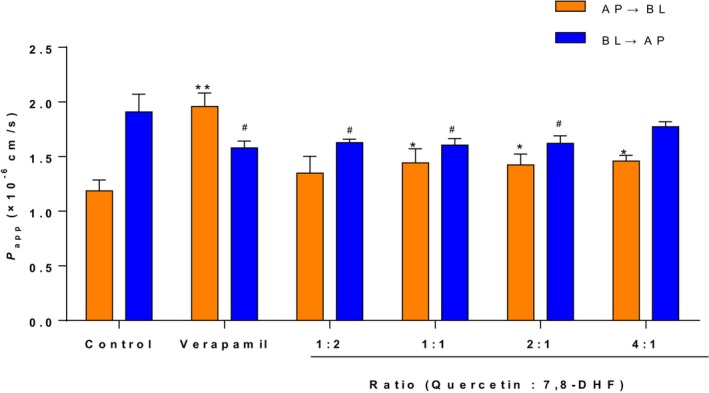
Effects of quercetin concentrations on bi‐directional *P*_app_ value of 7,8‐DHF across Caco‐2 monolayers. *p < .05, **p < .01 indicate a significant difference between each group and the control group in the AP → BL direction; ^#^ *p* < .05 indicates a significant difference between each group and the control group in the BL → AP direction.

**FIGURE 5:**
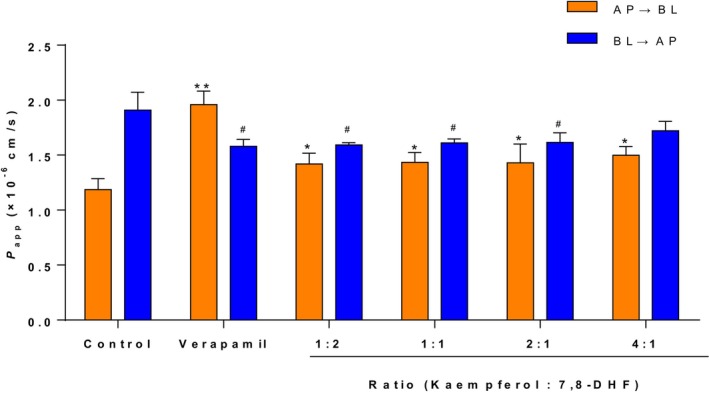
Effects of kaempferol concentrations on bi‐directional P_app_ value of 7,8‐DHF across Caco‐2 monolayers. *p < .05, **p < .01 indicate a significant difference between each group and the control group in the AP → BL direction; ^#^ *p* < .05 indicates a significant difference between each group and the control group in the BL → AP direction.

The effects of different concentrations of quercetin on the transport of 7,8‐DHF in Caco‐2 monolayer cell membranes are presented in Figure 4 and Table 2. The addition of varying concentrations of quercetin had varying impact on the transport of 7,8‐DHF compared with the control group, and when the molar concentration ratio of 7,8‐DHF to quercetin was 1:1, the ER value was the lowest (1.11). At this point, the *P*_app AB_ and *P*_app BA_ values of 7,8‐DHF were (1.44 ± 0.10) × 10^−6^ cm/s and (1.61 ± 0.05) × 10^−6^ cm/s, respectively. Similarly, the addition of different concentrations of kaempferol also had varying impact on the transport of 7,8‐DHF. However, with an increase in kaempferol concentration resulting in an increase in the ER value. When the molar concentration ratio of 7,8‐DHF to kaempferol was 2:1, the transport efficiency of 7,8‐DHF was the highest (Figure 5). At this point, the *P*_app AB_ and *P*_app BA_ values of 7,8‐DHF were (1.42 ± 0.08) × 10^−6^ cm/s and (1.59 ± 0.02) × 10^−6^ cm/s, respectively (Table 2). These findings indicate that different flavonoids may show differential impacts on the transport of 7,8‐DHF in Caco‐2 monolayer cell membranes, and the optimal ratio for enhancing transport efficiency may vary depending on the specific flavonoid compound (Fang et al., 2021). For example, *Zhou* et al. reported that EGCG and quercetin significantly increased the AP → BL influx and decreased the BL → AP efflux of acteoside across Caco‐2 monolayers with optimum molar ratio of EGCG to acteoside (1:1) and quercetin to acteoside (3:4; Zhou et al., 2020).

**TABLE 2:** Effects of quercetin and kaempferol concentrations on bi‐directional *P*_app_ and *ER* value of 7,8‐DHF across Caco‐2 monolayers.

Group	*P*_app AB_ (×10^−6^ cm/s)	*P*_app BA_ (×10^−6^ cm/s)	ER
Control	1.19 ± 0.08	1.91 ± 0.13	1.61
Verapamil	1.96 ± 0.10**	1.58 ± 0.05^#^	0.81
Quercetin: 7,8‐DHF = 1: 2	1.35 ± 0.12	1.63 ± 0.03^#^	1.21
Quercetin: 7,8‐DHF = 1: 1	1.44 ± 0.10*	1.60 ± 0.05^#^	1.11
Quercetin: 7,8‐DHF = 2: 1	1.42 ± 0.08*	1.62 ± 0.06^#^	1.14
Quercetin: 7,8‐DHF = 4: 1	1.46 ± 0.04*	1.77 ± 0.04	1.22
Kaempferol: 7,8‐DHF = 1: 2	1.42 ± 0.08*	1.59 ± 0.02^#^	1.11
Kaempferol: 7,8‐DHF = 1: 1	1.43 ± 0.07*	1.61 ± 0.03^#^	1.12
Kaempferol: 7,8‐DHF = 2: 1	1.43 ± 0.14*	1.62 ± 0.07^#^	1.13
Kaempferol: 7,8‐DHF = 4: 1	1.50 ± 0.06*	1.72 ± 0.07	1.15

*p < .05, **p < .01 indicates a significant difference between each group and the control group in the AP → BL direction; ^#^ *p*< .05 indicates a significant difference between each group and the control group in the BL → AP direction.

**FIGURE 6:**
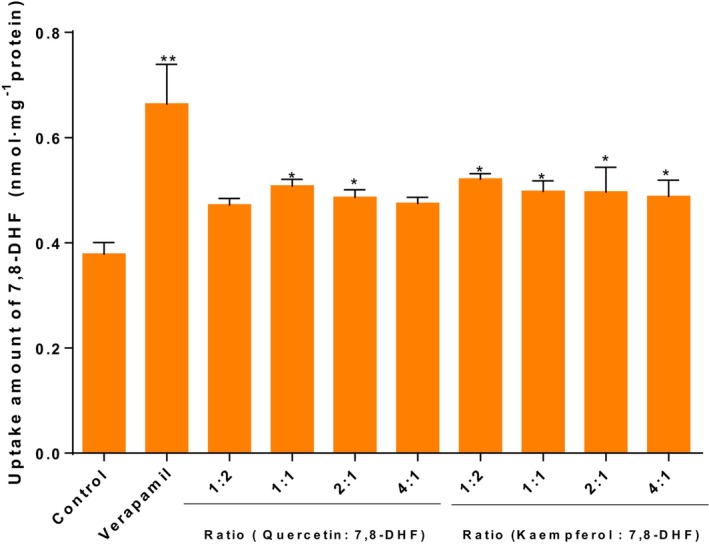
Uptake amount of 7,8‐DHF in the Caco‐2 cells at the end of transport time with various concentrations of flavonoids (nmol·mg^−1^ protein). *p < .05, **p < .01, versus Control, the significant differences were analyzed by unpaired two‐tailed Student's *t*‐test.

The impact of different concentrations of quercetin and kaempferol on the remaining amount of 7,8‐DHF in the cells was also investigated. As shown in Figure 6, various molar concentrations of kaempferol enhanced the uptake amount of 7,8‐DHF in Caco‐2 cells, especially when the molar concentration ratio of kaempferol to 7,8‐DHF was 1:2, resulting in a cell uptake amount of 7,8‐DHF of (0.521 ± 0.008) nmol·mg^−1^ protein. Conversely, quercetin did not increase the uptake of 7,8‐DHF in Caco‐2 cells when the molar concentration ratios of quercetin and 7,8‐DHF were 1:2 and 4:1, respectively. However, when the molar concentration ratio of quercetin to 7,8‐DHF was 1:1, the cell uptake amount of 7,8‐DHF reached the maximum value of (0.508 ± 0.011) nmol·mg^−1^ protein. Based on the results of different molar concentrations of kaempferol and quercetin on the transmembrane transport of 7,8‐DHF in Caco‐2 cells, they can be potentially utilized as P‐gp inhibitors when the molar concentration ratios of kaempferol and quercetin to 7,8‐DHF are 1:2 and 1:1, respectively.

### 3.5 Effect of flavonoid on structural and in vitro digestion stability of 7,8‐DHF

To evaluate the effect of intracellular enzymes on 7,8‐DHF, the stability of 7,8‐DHF in HBSS (pH = 7.4) and Caco‐2 cells with or without flavonoid inhibitors was measured. As described in Table S2, no significant difference in the percentage recovery of 7,8‐DHF in the Caco‐2 cells was observed compared with blank HBSS after 60 and 240 min incubation, indicating that 7,8‐DHF was not metabolized in the Caco‐2 cells. This was also reported by Xiao and Högger (2015), who found that the half‐degradation time of 7,8‐DHF was above 180 min in the DMEM solutions during Caco‐2 cell incubation. Additionally, the percentage recovery of 7,8‐DHF in the presence of different flavonoid inhibitors showed no significant difference compared with that in blank HBSS. Therefore, during the 150 min transport process, 7,8‐DHF remained stable. Furthermore, after the transport course of 7,8‐DHF with or without flavonoid inhibitors, the TEER values were all above 300 Ω cm^2^, indicating that the monolayers were not disrupted under different matrix environments (Table S1).

**FIGURE 7:**
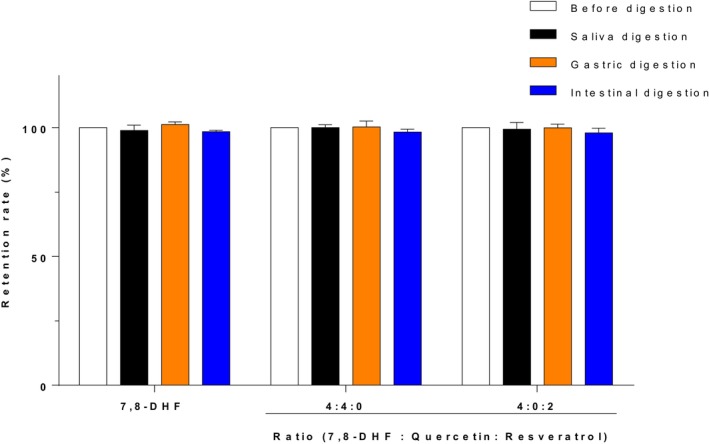
Retention rates of 7,8‐DHF during digestive system.

To assess the bioavailability of 7,8‐DHF after oral administration, it is crucial to investigate its stability in the gastrointestinal digestive system (Ajeeshkumar et al., 2021; Dai et al., 2020). Therefore, the anti‐digestive properties of 7,8‐DHF by simulating saliva, gastric juice, and intestinal fluid were also investigated. As demonstrated in Figure 7, the retention rates of 7,8‐DHF after digestion by saliva, gastric juice, and intestinal fluid were similar to those prior to digestion, indicating that digestive factors, such as enzymes, pH, and ion strength did not significantly degrade 7,8‐DHF. Additionally, when combined with two other flavonoids (quercetin and kaempferol) which have transport‐promoting effects, and subjected to simulated in vitro digestion, no significant effects were observed.

## 4 CONCLUSION

This study utilized an in vitro Caco‐2 monolayer cell model to investigate the effects of various types and concentrations of plant flavonoids on the transepithelial transport of 7,8‐DHF. Results indicated that quercetin and kaempferol significantly promoted the transepithelial transport of 7,8‐DHF by inhibiting its efflux, with the most effective inhibition of P‐gp efflux transport protein observed at molar concentration ratios of 1:1 and 2:1 for 7,8‐DHF to quercetin and kaempferol, respectively. Therefore, in the formulation of functional foods, quercetin, and/or kaempferol can be considered as synergistic enhancers for 7,8‐DHF. However, the inhibitory ability of flavonoids was relatively weak when compared with the specific P‐gp inhibitor‐positive control drug Verapamil. This indicates that screening P‐gp inhibitors from naturally derived plant flavonoids may have limited effects on promoting absorption and transport of 7,8‐DHF in intestinal epithelial cells, and further exploration of more effective methods and means to improve the bioavailability of orally administered 7,8‐DHF is needed. Additionally, results from simulated in vitro digestion experiments suggested that 7,8‐DHF maintains its structural stability throughout the entire digestive system. In future research, we will focus on investigating the impact of potential flavonoid compounds on the bioavailability of 7,8‐DHF in vivo.

## AUTHOR CONTRIBUTIONS

**Yufeng Chen:** Data curation (equal); funding acquisition (equal); methodology (equal); project administration (equal); resources (equal); writing – original draft (equal); writing – review and editing (equal). **Guobin Xia:** Conceptualization (equal); investigation (supporting); methodology (supporting). **Chunfeng Wang:** Data curation (supporting). **Huawei Wu:** Resources (equal); visualization (equal). **Xiaogang Xu:** Resources (supporting); software (supporting). **Genxiang Mao:** Formal analysis (supporting); writing – review and editing (supporting). **Jiong Wu:** Funding acquisition (equal); project administration (equal); supervision (equal). **Zhenlei Zhao:** Data curation (equal); funding acquisition (equal); project administration (equal); supervision (equal).

## FUNDING INFORMATION

The work was supported by the Zhejiang Province postdoctoral Merit‐based Research Project (grant number: ZJ2022033), the Zhejiang Province Public Welfare Technology Application Research Project‐National Cooperation Project (grant number: LGJ21C20001), the Zhejiang Medical and Health Science and Technology Plan Project (grant number: 2023KY440), the Project of the Research Center of Prevention and Treatment of Senescence Syndrome, School of Medicine Zhejiang University (grant number: 2022060002), the National Key Research and Development Program of China (grant number: 2022YFC3501802) and the Key project of China Administration of Traditional Chinese Medicine (grant number: GZY‐ZJ‐KJ‐23041).

## CONFLICT OF INTEREST STATEMENT

The authors declare that they have no known competing financial interests or personal relationships that could have appeared to influence the work reported in this paper.

## ETHICS STATEMENT

This study does not involve any human or animal testing.

## Supporting information


Tables S1‐S2

## Data Availability

Data will be made available on request.
