# Supplementary material for: Impact of dietary plant flavonoids on 7,8‐dihydroxyflavone transepithelial transport in human intestinal Caco‐2 cells
Source: Food Sci Nutr. 2023 Jul 30;11(11):6888–98. doi: 10.1002/fsn3.3581 (PMC10630842; doi:10.1002/fsn3.3581)
Supplement: Supplementary file 1 — Tables S1‐S2 [file FSN3-11-6888-s001.docx]

Impact of dietary plant flavonoids on 7,8-dihydroxyflavone transepithelial transport in human intestinal Caco-2 cells

Yufeng Chen^1,2,3^, Guobin Xia^4^, Chunfeng Wang^2^, Huawei Wu^3^, Xiaogang Xu^2^, Genxiang Mao^2^, Jiong Wu^2,*^, Zhenlei Zhao^2,*^

^1^*Department of Food Science and Nutrition, School of Biosystems Engineering and Food Science, Zhejiang Key Laboratory for Agro-Food Processing; Zhejiang Engineering Center for Food Technology and Equipment; Zhejiang University, Hangzhou 310058, China*

^2^*Zhejiang Provincial Key Lab of Geriatrics & Geriatrics Institute of Zhejiang Province, Department of Geriatrics, Zhejiang Hospital, Hangzhou 310030, China*

^3^*Ningbo Today Food Co Ltd, Ningbo, 315000, China*

^4^*Section of Neonatology, Department of Pediatrics, Baylor College of Medicine, Houston, TX 77030, USA*

* Corresponding author:

*Zhejiang Provincial Key Lab of Geriatrics & Geriatrics Institute of Zhejiang Province, Department of Geriatrics, Zhejiang Hospital, Hangzhou 310030, China*

E-mail addresses: wujiong0118@aliyun.com (J. Wu); zhenlei@zju.edu.cn (Z.Zhao)

**Supplement Table 1** The transepithelial electrical resistance (TEER) values (Ω•cm^2^) before and after transport experiments (Mean ± SD, n = 3)

| Group | AP→BL | | BL→AP | |
| --- | --- | --- | --- | --- |
|  | Before After | | Before After | |
| 7,8-DHF | 368.8±13.0 | 353.5±7.66 | 349.2±3.75 | 333.3±5.52 |
| 7,8-DHF+ Verapamil | 327.3±17.2 | 308.4±3.56 | 335.6±18.9 | 314.1±5.95 |
| 7,8-DHF+ Hesperetin | 339.9±21.0 | 326.2±24.7 | 336.9±10.9 | 312.9±3.31 |
| 7,8-DHF+ Naringenin | 363.3±15.6 | 336.9±18.6 | 367.6±16.0 | 325.7±16.3 |
| 7,8-DHF+ EGCG | 372.9±19.6 | 346.6±11.7 | 351.9±21.9 | 337.2±20.5 |
| 7,8-DHF+ Baicalein | 405.9±12.9 | 340.9±27.3 | 355.9±26.1 | 316.3±7.54 |
| 7,8-DHF+ Fisetin | 339.9±22.9 | 310.1±8.31 | 323.3±8.90 | 311.9±7.54 |
| 7,8-DHF+ Kaempferol | 337.6±36.7 | 312.5±13.3 | 387.9±18.4 | 368.2±11.8 |
| 7,8-DHF+ Biochanin A | 331.9±8.62 | 315.3±10.3 | 339.5±16.9 | 312.5±15.4 |
| 7,8-DHF+ Quercetin | 350.8±22.1 | 322.0±14.9 | 409.9±28.0 | 366.0±31.6 |
| 7,8-DHF+ Myricetin | 331.1±10.7 | 321.9±10.7 | 384.0±17.3 | 324.3±12.3 |
| 7,8-DHF+ Genistein | 351.8±19.6 | 329.5±15.3 | 337.3±18.7 | 319.6±14.3 |
| 7,8-DHF+ Orientin | 400.93±24.3 | 346.2±31.0 | 352.2±27.8 | 323.2±17.5 |
| 7,8-DHF+ Isoorientin | 349.3±21.9 | 322.0±16.6 | 333.8±26.6 | 321.2±16.4 |
| 7,8-DHF+ Vitexin | 334.9±22.1 | 317.6±17.8 | 410.7±23.2 | 318.9±13.6 |
| 7,8-DHF+ Isovitexin | 321.3±8.20 | 311.4±7.84 | 340.0±26.9 | 374.2±18.7 |
| 7,8-DHF+ Kaempferol （molar ratios 2:1） | 353.2±29.9 | 333.3±25.9 | 370.8±12.3 | 325.3±16.8 |
| 7,8-DHF+ Kaempferol  （molar ratios 1:2） | 338.6±21.4 | 320.9±16.5 | 351.2±24.4 | 326.9±19.1 |
| 7,8-DHF+ Kaempferol  （molar ratios 1:4） | 400.83±26.5 | 355.2±22.8 | 358.2±19.3 | 326.9±13.0 |
| 7,8-DHF+ Quercetin  （molar ratios 2:1） | 338.3±27.7 | 319.9±25.7 | 392.3±26.4 | 348.9±17.0 |
| 7,8-DHF+ Quercetin  （molar ratios 1:2） | 335.2±26.4 | 316.6±12.2 | 446.6±39.3 | 326.5±29.4 |
| 7,8-DHF+ Quercetin  （molar ratios 1:4） | 350.2±27.5 | 337.2±20.4 | 443.6±38.1 | 330.9±17.9 |

**Supplement Table 2** Percentage recovery of 7,8-DHF at the different incubation time in HBSS and Caco-2 cells with or without inhibits (Mean ± SD, n = 3)

| Group | Recovery of 7,8-DHF (%) | |
| --- | --- | --- |
|  | 60 min | 240 min |
| HBSS | 98.71±1.07 | 97.26±2.17 |
| HBSS (Caco-2) | 99.11±0.57 | 97.74±0.42 |
| HBSS (Caco-2)+ Verapamil | 98.32±1.42 | 96.58±1.98 |
| HBSS (Caco-2)+ Hesperetin | 99.12±0.67 | 96.19±3.00 |
| HBSS (Caco-2)+ Naringeni | 99.08±0.64 | 96.58±2.53 |
| HBSS (Caco-2)+ EGCG | 99.03±0.53 | 96.62±2.55 |
| HBSS (Caco-2)+ Baicalein | 98.32±1.10 | 96.18±3.02 |
| HBSS (Caco-2)+ Fisetin | 98.15±1.46 | 97.59±1.73 |
| HBSS (Caco-2)+ Kaempferol | 99.00±1.18 | 97.28±0.79 |
| HBSS (Caco-2)+ Biochanin A | 98.58±1.09 | 97.28±0.76 |
| HBSS (Caco-2)+ Quercetin | 99.24±0.74 | 97.90±0.92 |
| HBSS (Caco-2)+ Myricetin | 98.57±0.87 | 97.62±1.69 |
| HBSS (Caco-2)+ Genistein | 99.12±0.60 | 96.51±1.69 |
| HBSS (Caco-2)+ Orientin | 98.50±1.57 | 97.44±2.56 |
| HBSS (Caco-2)+ Isoorientin | 98.34±1.07 | 96.62±1.70 |
| HBSS (Caco-2)+ Vitexin | 98.21±1.49 | 97.40±2.95 |
| HBSS (Caco-2)+ Isovitexin | 99.01±1.11 | 97.12±1.97 |
| HBSS (Caco-2)+ Kaempferol  （molar ratios 2:1） | 98.97±0.45 | 97.80±1.82 |
| HBSS (Caco-2)+ Kaempferol  （molar ratios 1:2） | 98.08±1.43 | 96.73±2.18 |
| HBSS (Caco-2)+ Kaempferol  （molar ratios 1:4） | 98.78±1.06 | 97.32±0.90 |
| HBSS (Caco-2)+ Quercetin  （molar ratios 2:1） | 98.74±0.79 | 97.31±0.79 |
| HBSS (Caco-2)+ Quercetin  （molar ratios 1:2） | 98.70±1.07 | 96.77±2.40 |
| HBSS (Caco-2)+ Quercetin  （molar ratios 1:4） | 99.33±0.22 | 98.04±0.60 |
